# Uveitis in Children: The Role of Biological Agents in Its Management

**DOI:** 10.3390/biomedicines11020629

**Published:** 2023-02-20

**Authors:** Jamel Corredores, Brice Vofo, Radgonde Amer

**Affiliations:** 1 Department of Ophthalmology, Hadassah Medical Center, Jerusalem 91120, Israel; 2 Faculty of Medicine, The Ophthalmology Department, Hadassah Medical Center, Hebrew University of Jerusalem, Jerusalem 91120, Israel

**Keywords:** pediatric uveitis, juvenile idiopathic arthritis, TNF-α blockers, adalimumab, infliximab

## Abstract

We aimed to determine medium and long-term effects of TNF-α inhibitors in patients with pediatric uveitis. This was a retrospective review of medical charts. Included were 50 patients (84 eyes). Mean age at diagnosis was 7.22 ± 4.04 years. At baseline (time of initiation of biologic therapy), all patients had active uveitis. Complete control of uveitis was achieved in 84.52% (*n* = 71) of eyes, after a median of 3 months (IQR 2 months). Mean LogMAR BCVA at baseline was 0.23 ± 0.44; it remained stable at 12 and 24 months. At baseline, 64% of patients were treated with oral corticosteroids, this decreased to 29.5% at 12 months (*p* = 0.001) and to 21.9% at 24 months (*p* < 0.001). Mean time to prednisone dose of ≤0.2 mg/kg/day was 8.1 ± 2.02 months after baseline. A total of 40.5% of eyes were treated with topical steroids at baseline and this significantly decreased to 5.8% at 12 months. Multiple linear regression model was calculated to predict moderate and severe visual loss; only presenting visual acuity accounted for a unique variance in the model. In conclusion, TNF-α inhibitors achieved rapid disease control while enabling a remarkable steroid-sparing effect in children suffering from chronic uveitis. Presenting visual acuity was the sole predictor of moderate to severe visual loss.

## 1. Introduction

Uveitis in children is less common than in adults and it is usually characterized by being chronic [1]. The smoldering course of pediatric uveitis and the subsequent delay in seeking medical attention represent factors that eventually lead to the high occurrence of ocular complications at presentation. In a study by Habot-Wilner et al. [1] on a cohort of 107 children (182 eyes) with pediatric uveitis, 74% had ocular complications at presentation. Similarly, de Boer et al. [2] reported on ocular complications in 76% of patients in a cohort of 123 patients with pediatric uveitis. This fact, in addition to the risk of visual impairment early in life, requires ophthalmologists to choose the administration of the most efficient treatment strategies, yet with the least possible systemic and ocular adverse effects. Corticosteroids, despite being potent anti-inflammatory medications, are not favored in children because of the plethora of associated side effects, particularly growth retardation and osteoporosis. Steroid-sparing agents, especially methotrexate (MTX), are in wide use for pediatric inflammatory conditions. MTX is the most widely used disease-modifying anti-rheumatic drug in the treatment of juvenile idiopathic arthritis (JIA) [3], owing to its efficacy, its relatively safe profile, to the weekly administration and the option of subcutaneous use rather than oral delivery which may be particularly challenging in the young.

Biological agents have revolutionized the treatment of systemic and ocular inflammatory conditions. In the Sycamore study [4] adalimumab controlled inflammation and resulted in a lower rate of treatment failure than placebo among children and adolescents with active JIA-associated uveitis. The VISUAL studies [5,6] have investigated the role of adalimumab in adults with non-infectious intermediate, posterior or panuveitis (NIPPU). However, few publications exist on the use of TNF-α inhibitors in children with uveitis.

The aim of the present study was to examine the medium and long-term effects of TNF-α inhibitors in children with chronic uveitis unresponsive to conventional immunomodulatory treatment (IMT). In addition, we aimed to study the visual outcome among patients with anterior vs. non-anterior uveitis and JIA-related vs. non-JIA-related uveitis. 

## 2. Materials and Methods

This was a retrospective longitudinal study of a consecutive group of patients suffering from chronic refractory noninfectious uveitis and treated with TNF-α inhibitors. The study included children ≤18 years of age, who failed to respond to treatment with corticosteroids and a conventional IMT with at least 3 months of follow-up after baseline. Baseline was defined as the time when anti-TNF-α therapy was started. Adalimumab (Humira®; AbbVie Inc., Ludwigshafen, Germany), was instituted at a dose of 40 mg subcutaneously and repeated every other week in patients weighing ≥30 kg. In children weighing ˂30 kg, adalimumab was instituted at a dose of 20 mg subcutaneously and repeated every other week. Infliximab (Remicade®; Merck & Co., Inc., NJ, USA) (5 mg/kg intravenously) was administered at baseline, at weeks 2 and 6, and every 4–6 weeks afterwards (adalimumab has a mean terminal half-life of approximately 2 weeks while infliximab has a serum half-life of 9.5 days). All the patients were on a stable dose of steroid-sparing agents for at least 12 weeks before baseline. The study was approved by the institutional review board and included waived informed consent for the chart review. The study was conducted in adherence to the tenets of the declaration of Helsinki.

Complete blood counts, liver and kidney function tests, serological tests for hepatitis B and hepatitis C, Mantoux tests ± QuantiFERON tests and chest radiographs were obtained before baseline. Brain magnetic resonance imaging was performed to rule out demyelinating disease in patients with intermediate uveitis.

Demographic and clinical data that were collected included gender, age at diagnosis of uveitis, anatomical diagnosis according to the Standardization of Uveitis Nomenclature (SUN) group criteria [7], associated systemic diseases, follow-up and types of IMT.

Snellen’s best-corrected visual acuity (BCVA) was recorded at baseline and at 3, 6, 9, 12 and 24 months after baseline. LogMAR (logarithm of the minimum angle of resolution) notation was used to compute the change in VA. Moderate vision loss was defined as BCVA of ≤20/50 and >20/200 and severe vision loss was defined as BCVA ≤20/200. Ocular complications causing moderate and severe vision loss were collected. 

Response to treatment, time to first flare, rate of flares in the year preceding baseline and in the first year following baseline, the dose of systemic corticosteroids before and after baseline, and time lapse to prednisone dose of ≤0.2 mg/kg/day were documented.

Disease control was defined as the time to first flare after baseline being more than 6 months [8]. Flare was defined as evidence of worsened intraocular inflammation occurring in the treated eyes at least 6 months after inflammation was controlled, and required reintroduction or addition of another IMT [8] or escalation of the dose of systemic corticosteroids. We followed the SUN definition of worsening activity [7]. Treatment failure was defined as repeat flares (≥2) or systemic side effects and resulted in stopping or switching the biological drug [8].

## 3. Statistical Analysis

Statistical analyses were performed using SPSS Statistics, version 25.0 (IBM Corp., Armonk, NY, USA). Frequency counts and percentages were generated where appropriate. Tests of normality were performed on the data, and parametric and non-parametric tests were applied as needed. Linear regression was used to model the probability of the occurrence of binary dependent variables (risk factors for mild or no visual loss vs. moderate and severe visual loss). A multiple linear regression model was calculated in order to predict the risk factors for moderate and severe visual loss based on significant factors seen in univariate analysis. Statistical significance was defined as a *p*-value < 0.05.

## 4. Results

### 4.1. Demographic Characteristics, Associated Systemic Diseases and Types of Uveitis

Included were 50 patients (84 eyes), of whom 35 were females (70%). Mean age at time of diagnosis of uveitis was 7.22 ± 4.04 years (range 2–18). At baseline, all patients had active uveitis. It was anterior in 51 eyes (60.71%), intermediate in 19 eyes (22.61%), and posterior and panuveitis in 7 eyes each (8.33%). Ocular disease was bilateral in 34 (68%) patients. Mean total follow-up time was 4.86 ± 2.82 years (range 1–11 years) and mean follow-up time after baseline was 38.62 ± 31.29 months (range 3–120 months). Twenty-five (50%) patients had follow-up longer than 30 months (Table 1).

Thirty-one patients (62%) had an associated systemic disease; of those, JIA was disclosed in 28 patients (90.32%), whereas psoriasis, sarcoidosis and Behçet disease were diagnosed in one patient each (3.22%).

The indication to start biological treatments was active ocular disease due to partial or no response to IMT. Three patients (6%) had additionally active systemic diseases which necessitated the initiation of biological agents. No child was found not to qualify for biological therapy because of abnormal results of blood tests or imaging studies.

### 4.2. Efficacy of TNF-α Inhibitors

Complete control of uveitis was achieved with the first biological agent in 71 eyes (84.52%) of 41 patients, after a median of 3 months (IQR 2 months).

Adalimumab was used in 45 patients (90%) and infliximab in 5 patients (10%). Median time to introduction of biological agents after institution of conventional IMT was 10 months (IQR 12 months).

The median number of flares/eye in the year preceding the institution of anti-TNF-α therapy was one flare/year (IQR 1 flare/year) and it decreased to 0 flares/year (IQR 0 flares/year) in the first year after baseline (*p* < 0.0001).

Twenty eyes (23.8%) experienced flares after a mean of 10.07 ± 11.72 months (range 5 to 24 months); 15 eyes were being treated with adalimumab and 5 eyes with infliximab.

### 4.3. Steroid-Sparing Effect

**Figure 1:**
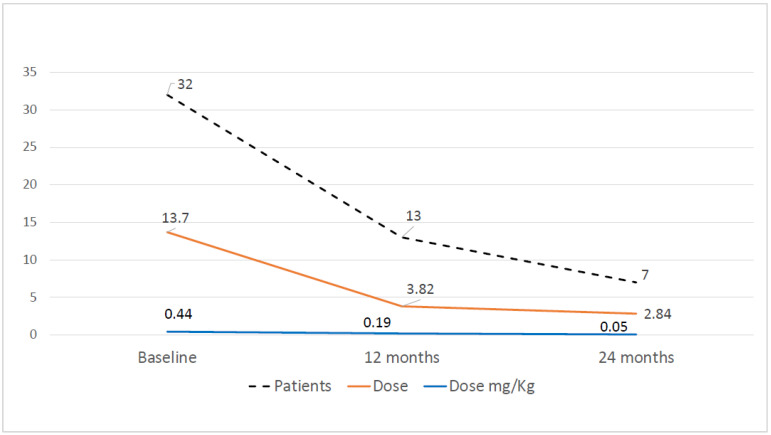
Number of patients who were treated with prednisone, dose of prednisone in mg/day and in mg/kg at baseline and at 12 months and 24 months after baseline.

The mean initial dose of prednisone was 13.70 ± 13.47 mg/day (mean 0.44 mg/kg ± 0.41) and it decreased to 3.82 ± 3.02 mg/day (mean 0.19 ± 0.24 mg/kg) (*p* < 0.0001) and 2.84 ± 2.81 mg/day (mean 0.05 ± 0.06 mg/kg) (*p* < 0.0001 compared to baseline) at 12 and 24 months, respectively. At baseline, 32 patients (64%) were dependent on oral corticosteroids, this decreased to 13 patients (29.5%) at 12 months (*p* = 0.001) and to 7 patients (21.9%) at 24 months (*p* < 0.001 compared to baseline) (Figure 1). Mean time to prednisone dose of ≤0.2 mg/kg/day was 8.1 ± 2.02 months after baseline.

**Figure 2:**
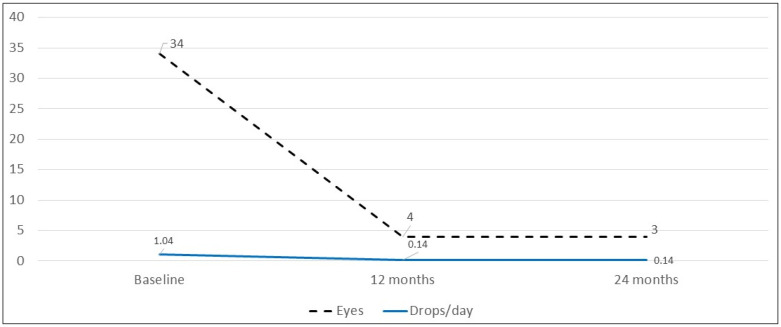
Number of eyes that were treated with topical steroids and the mean dose of topical steroids (drops/day) at baseline and at 12 months and 24 months after baseline.

At baseline, 34 (40.48%) eyes were treated with topical steroids. This decreased to 4 eyes (5.8%) at 12 months (*p* < 0.0001) and remained stable at 24 months (3 eyes, 4.5%). Median dose of topical steroids decreased from 1.5 drops/day (IQR 1.25) at baseline to a median dose of 0.0 drops/day (IQR 0) at month 12 and remained stable at 24 months (*p* < 0.0001 in each case) (Figure 2).

Of all eyes that used topical steroids at baseline (*n* = 34), five eyes (14.7%) used topical corticosteroids >3 drops daily. No eyes received topical corticosteroids >3 drops daily at 12 and 24 months (*p* = 0.053).

### 4.4. Immunomodulatory Therapy

At baseline, 47 patients (94%) used one IMT drug, of those 42 (89.37%) used MTX and 5 (10.63%) used azathioprine. At 12 and 24 months, 38 (out of 44) patients (82.60%) and 29 patients (out of 32) (90.62%) still used one type of second-line IMT, respectively.

### 4.5. Effect on Visual Acuity

**Figure 3:**
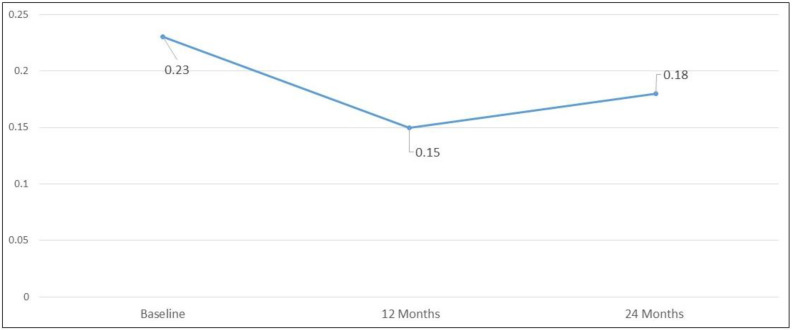
Mean LogMAR best-corrected visual acuity at baseline and at 12 and 24 months after baseline.

**Figure 4:**
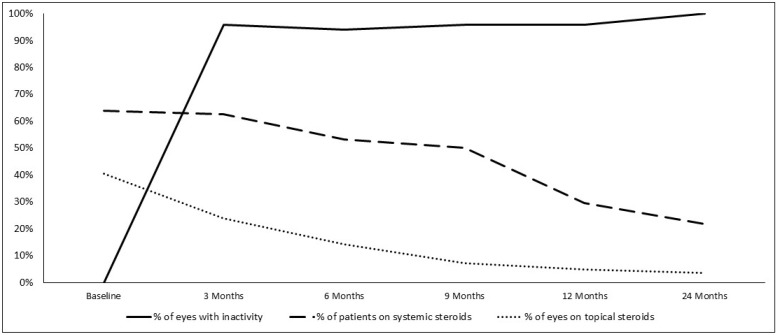
Number of eyes with inactivity, number of patients on systemic steroids and number of eyes on topical steroids between baseline and 24 months later.

Mean initial logMAR BCVA was 0.23 ± 0.44; it remained stable at 0.15 ± 0.40 (*p* = 0.63) and 0.18 ± 0.41 (*p* = 0.610) at 12 and 24 months, respectively (Figure 3 and Figure 4).

At baseline, 74 eyes (88.10%) had BCVA ≥20/40, 6 eyes (7.14%) had moderate vision loss and 4 eyes (4.76%) showed severe vision loss. The percentage of eyes with BCVA ≥20/40 remained stable at 12 and 24 months after baseline (60/69 eyes) (86.95%) and 57/66 eyes (86.36%), respectively) (*p* = 1.0 and *p* = 0.808, respectively). The rate of severe visual loss (BCVA ≤ 20/200) remained stable at all time points: (2/69 eyes) (2.9%) at 12 months and 2/66 eyes (3.03%) at 24 months (*p* = 0.436 and *p* = 0.695, respectively).

### 4.6. Ocular Complications and Treatment Failure

Treatment failure occurred in six patients (12%): two were treated with adalimumab and four with infliximab. The two patients who were treated with adalimumab failed treatment at 7 and 60 months after baseline and the four patients who were treated with infliximab failed treatment at 12, 28, 60 and 72 months after baseline. 

Cataract and band keratopathy were the most common complications at baseline, each was diagnosed in 14 eyes (16.7%). Cystoid macular edema (CME) was the second most common complication, diagnosed in 11 eyes (13%). CME resolved at a mean of 3.81 ± 1.89 months following the institution of TNF-α inhibitors. Additional infrequent complications at baseline included glaucoma in 3 eyes, vitreous hemorrhage in 2 eyes, and optic disc neovascularization in 1 eye. At 24 months after baseline, 9 eyes of 9 patients had BCVA <20/40, all of which suffered from anterior uveitis which was JIA-related in 8 patients and secondary to sarcoid-related uveitis in the 9th patient. At the last follow-up, the complications that resulted in severe visual loss were macular atrophy and optic atrophy, coexisting in 2 eyes.

Surgical intervention for the management of ocular complications was required in 7 eyes of 6 patients: one patient underwent pars plana vitrectomy for non-resolving vitreous hemorrhage (1 eye); 5 patients underwent lensectomy and vitrectomy due to dense cataract impeding fundus visualization (6 eyes).

### 4.7. Multiple Regression Model

Presenting visual acuity, duration of the disease before starting anti-TNF-α therapy, type of anti-TNF-α drug, anatomical type of uveitis and etiological type of uveitis were the variables included in univariate analysis of predictors of moderate to severe visual loss. Neither anatomical type of uveitis (anterior vs. non-anterior) nor etiological type of uveitis (JIA-associated vs. non JIA-associated) were associated with moderate to severe visual loss (*p* = 0.279 and *p* = 0.077, respectively). However, better visual acuity at presentation and the use of adalimumab as first-line biological drug correlated inversely with the occurrence of moderate and severe visual loss (Spearman’s rho 485 and 333; *p* < 0.0001 and *p* = 0.018, respectively). In addition, there was an inverse correlation between the disease duration before biologics and the occurrence of moderate to severe visual loss, (Spearman’s rho 472, *p* < 0.001).

Hence, a multiple linear regression model was calculated to predict moderate and severe visual loss based on presenting visual acuity, duration of the disease before starting anti-TNF-α therapy and type of anti-TNF-α drug used as first-line. The overall regression model was significant (F(3,45):8.56, R^2^ = 0.36, *p* < 0.001). However, only presenting visual acuity accounted for a unique variance in the model (*p* < 0.001), while duration of the disease before starting anti-TNF-α therapy and type of anti-TNF-α drug used as first-line did not significantly account for a unique variance in the model (*p* = 0.138 and *p* = 0.624, respectively). Therefore, presenting visual acuity was the sole predictor of moderate to severe visual loss, amongst the variables analyzed.

## 5. Discussion

The results of the present study demonstrated that TNF-α inhibitors achieved rapid control of pediatric uveitis while enabling a remarkable systemic and topical corticosteroid-sparing effect. Presenting visual acuity was the sole predictor of moderate to severe visual loss.

TNF-α inhibitors enabled complete control of uveitis after a median of 3 months. Similarly, Kouwenberg et al. [9] reported that disease inactivity with adalimumab was achieved in 91% of children after a median of three months. Such a prompt response is essential, particularly in eyes that present with sight-threatening complications. In the latter study, 65% of children had non-anterior uveitis while in the present study, 60.71% of eyes had anterior uveitis.

This quick control of inflammation subsequently resulted in rapid steroid taper. The percentage of patients using systemic corticosteroids dropped by more than half in the first year after the introduction of the biological agents. This persistent steroid-sparing effect allowed more patients to discontinue systemic corticosteroids in the second year. Despite the multitude of corticosteroid-related side-effects, clinicians prescribe them in cases where the potential benefits outweigh the potential risks. Additionally, corticosteroid bursts [10] used to treat acute exacerbations of inflammatory and allergic conditions, were found to be associated with a 1.4- to 2.2-fold increased risk of gastrointestinal bleeding, sepsis and pneumonia within the first month after their initiation.

Kouwenberg et al. [9] reported that the median initial dose of systemic corticosteroids was 10 mg/day, and it dropped to 0 mg/day 24 months later. Similarly, in the current study, the mean initial dose of prednisone was 13.70 ± 13.47 mg/day and it was reduced to 2.84 ± 2.81 mg/day, 24 months later. This steroid-sparing effect was achieved relatively fast as the mean time to a prednisone dose of ≤0.2 mg/kg/day was 8.1 ± 2.02 months after baseline.

Aiming to decrease and discontinue systemic steroids is an important milestone because of the plethora of side-effects which may negatively affect the child’s physical and psychological well-being. The disease itself in children exerts academic, interpersonal, psychological and developmental challenges [11]. Both the disease and its treatment affect quality of life. Adalimumab in pediatric Crohn’s disease was reported to induce and maintain clinical remission and improve growth and quality of life by achieving steroid-sparing remission [12].

Although we used topical steroids less frequently than Kouwenberg et al. [9] in their study, in both studies there was a markedly reduced dependence on topical steroids after baseline: in the current study, the median dose of topical steroids decreased from 1.5 drops/day (IQR 1.25) at baseline to median dose of 0.0 drops/day (IQR 0) at month 12. Kouwenberg et al. [9] reported that the median initial dose of topical steroids was 2 drops/day and this decreased to 0.6 drops/day at 24 months. In the latter study, 33.3% of eyes were treated with >3 drops of topical steroids daily at baseline and this decreased to 6.9% at 24 months. In the current study, of the 34 eyes that were treated with topical steroids at baseline, 30 eyes (88%) were free of topical steroids at 12 months and no eyes received topical corticosteroids >3 drops daily at 12 and 24 months.

Ophthalmologists aim to withhold the use of topical steroids in children with chronic uveitis because of the potential ocular complications that may ensue. Cataract was the most common complication at presentation in the current study. Additionally, it was reported to be a common complication of pediatric uveitis in other studies [1,13]. Kouwenberg et al. [9] reported that 39.5% of their patients had cataract requiring surgery. Thorne et al. [14] investigated the risk of cataract development among patients with JIA-associated uveitis. The authors described a dose-dependent increase in the rate of cataract development among eyes receiving topical corticosteroids. Treatment with <3 drops daily of topical corticosteroid was associated with an 87% lower risk of cataract formation compared with eyes treated with >3 drops daily. Since persistent uveitis is a risk factor for cataract development, avoiding cataract progression by limiting topical steroid use is our responsibility, particularly in the pediatric age group in which the surgical outcomes may be guarded in some cases.

Children are especially susceptible to an IOP increase with steroid use [15]. Gupta et al. [16] reported a prevalence of 4.7% (59 children) with steroid-induced glaucoma among 1259 cases with pediatric glaucoma. The majority of those children (87%) were prescribed topical steroids for vernal keratoconjunctivitis and half of them had been on topical steroids for >1 year. Glaucomatous optic neuropathy was the cause of blindness in 37.3% (22/59), and 27% (16/59) were unilaterally blind at presentation. 

Kouwenberg et al. [9] reported an improvement in visual acuity from logMAR BCVA of 0.16 ± 0.55 at baseline to 0.05 ± 0.19 at 24 months. In the current study, logMAR BCVA at baseline was 0.23 ± 0.44 and it remained stable at 0.18 ± 0.41 at 24 months. The difference in visual acuities between the two studies and the lack of improvement in the current study could be attributed to the fact that the current study included eyes with JIA-associated uveitis and included a higher percentage of anterior uveitis than the former study (60.71% vs. 35%). Anterior uveitis in children is usually asymptomatic and commonly associated with ocular complications at presentation. Additionally, Kouwenberg et al. [9] reported that 28% of eyes had no active inflammation at baseline whereas all of our patients had active uveitis at baseline. These factors could have led to worse presenting BCVA in the present cohort and eventually patients sustained the same level of vision during the follow-up.

As presenting visual acuity was the sole predictor of moderate to severe visual loss and because many children with uveitis are asymptomatic [1,17], performing screening eye exams for children is of utmost importance in order to disclose treatable conditions such as uveitis while children still enjoy good visual acuity.

Kouwenberg et al. [9] disclosed that boys achieved disease inactivity faster than girls with a hazard ratio (HR) of 3.34 (95% CI 1.54–7.27, *p* = 0.002). The authors however did not elaborate on the reason that could account for this positive association between male gender and inactivity of uveitis. Furthermore, during early rheumatoid arthritis (RA), several cohort studies revealed that remission rates were lower for females than males treated with TNF-inhibitor therapies [18,19,20,21,22,23]. Male sex was found to be an independent predictor of sustained clinical remission in early RA patients on TNF inhibitors [18]. To date, no studies have addressed the mechanisms behind why immunotherapies, including TNF inhibitors, have greater efficacy and fewer adverse reactions in males compared with females.

This study was limited by its retrospective design and the variability of the follow-up schedules. Nonetheless, all data were structurally noted in the electronic patient files thus allowing a uniform way of registration and data collection. This cohort represents additional evidence on the role that biologic therapy plays in the context of pediatric uveitis. We have to keep in mind that children treated with TNF-α inhibitors remain at a greater risk of adverse events than patients treated with conventional IMT. The sycamore study (that evaluated the efficacy of adalimumab in JIA-associated uveitis patients taking a stable dose of MTX) showed that patients who received adalimumab had a much higher incidence of adverse events and serious adverse events than those who received placebos. The most common adverse events in the adalimumab group were minor infections, respiratory disorders and gastrointestinal disorders [3].

In conclusion, the present study further enhances our knowledge on the usefulness of TNF-α blockers in children with chronic refractory uveitis. It presents evidence on the effectivity of biological therapy, on the prompt control of uveitis and on the steroid-sparing effect while preserving vision. 

## Figures and Tables

**Table 1:** Demographics, anatomical and etiological types of uveitis, follow-up and biological agents used.

**Patients (eyes)**	50 (84)
**Age at diagnosis**	7.22 ± 4.04 years (range 2–18 years)
**Gender**	70% females (35), 30% males (15)
**Laterality**	68% bilateral (34 patients), 32% unilateral (16 patients)
**Anatomical types of uveitis**	84 eyes
**(number of eyes/%)**	
Anterior	51 (60.7%)
Intermediate	19 (22.6%)
Posterior	7 (8.3%)
Panuveitis	7 (8.3%)
**Systemic associations**	31 patients
**(number of patients/%)**	
Juvenile-associated uveitis	28 (90.3%)
Psoriasis	1 (3.2%)
Sarcoidosis	1 (3.2%)
Behçet disease	1 (3.2%)
**Total length of follow-up**	4.86 ± 2.82 years (range 1–11 years)
**Length of follow- up after baseline**	38.62 ± 31.29 months (range 3–120 months)
**Biological agent used**	Adalimumab 90% (45 patients), infliximab 10% (5 patients)

## Data Availability

Not applicable.
